# Supplementary material for: The global spectrum of tree crown architecture
Source: Nat Commun. 2025 May 26;16:4876. doi: 10.1038/s41467-025-60262-x (PMC12106748; doi:10.1038/s41467-025-60262-x)
Supplement: Supplementary file 1 — Supplementary Information [file 41467_2025_60262_MOESM1_ESM.pdf]

Supplementary Table 1 – Tree allometry database summary

Supplementary Table 1: Number of species and trees that underpin the analyses presented in the main text. Environmental predictors included in the models were tree cover, aridity, rainfall seasonality, mean annual temperature, wind gust speed and burned area (see Supplementary Table 2 for details on data sources). To test for phylogenetic signal, only species that directly matched those in the Smith & Brown (2018) phylogeny of seed plants were included.

	Tree height		Crown diameter		Crown aspect ratio	
**Analysis**	**Species**	**Trees**	**Species**	**Trees**	**Species**	**Trees**
Environmental	1910	373,666	1313	252,950	1309	251,733
Environmental + wood density	1572	338,925	1063	227,554	1059	226,390
Environmental + leaf nitrogen	1085	289,427	749	198,965	747	197,951
Environmental + SLA	1120	304,518	715	207,187	713	206,239
Environmental + seed mass	1108	311,883	703	206,347	701	205,240
Phylogenetic signal	1225	261,218	870	174,716	868	174,683

Supplementary Table 2 – Climate, tree cover, disturbance and biome data sources

Supplementary Table 2: Sources from which data on climate, tree cover, disturbance and biome classification were obtained for this study.

Environmental layer	Units	Resolution	Format	Source
Mean annual temperature (MAT)	°C	30 arc-seconds	Raster	https://www.worldclim.org/data/worldclim21.html
Maximum temperature warmest month	°C	30 arc-seconds	Raster	https://www.worldclim.org/data/worldclim21.html
Minimum temperature coldest month	°C	30 arc-seconds	Raster	https://www.worldclim.org/data/worldclim21.html
Temperature seasonality	°C	30 arc-seconds	Raster	https://www.worldclim.org/data/worldclim21.html
Mean annual precipitation (MAP)	mm	30 arc-seconds	Raster	https://www.worldclim.org/data/worldclim21.html
Precipitation seasonality	mm	30 arc-seconds	Raster	https://www.worldclim.org/data/worldclim21.html
Potential evapotranspiration (PET)	mm	30 arc-seconds	Raster	https://csidotinfo.wordpress.com/2019/01/24/global-aridity-index-and-potential-evapotranspiration-climate-database-v3/
Aridity index (PET/MAP)	unitless	30 arc-seconds	Raster	https://csidotinfo.wordpress.com/2019/01/24/global-aridity-index-and-potential-evapotranspiration-climate-database-v3/
Tree cover	%	15 arc-seconds	Raster	https://globalmaps.github.io/ptc.html
Wind gust speed [a]	$m\ s^{-1}$	5 arc-minutes	Raster	https://www.ecmwf.int/en/era5-land
Burned area fraction [b]	%	15 arc-minutes	Raster	https://www.globalfiredata.org/index.html
Snow cover duration [c]	$days\ yr^{-1}$	15 arc-seconds	Raster	https://download.geoservice.dlr.de/GSP/files/yearly/SCD/
Terrestrial biome classification [d]	7 classes†		Vector	https://www.worldwildlife.org/publications/terrestrial-ecoregions-of-the-world

[a] Maximum hourly wind speed between 2010-2020 estimated from ERA5-Land data

[b] Mean burned area fraction between 2001-2010 estimated from MODIS

[c] Maximum snow cover duration between 2001-2021 estimated from MODIS

[d] The Terrestrial Ecoregions of the World database groups different regions of the world into 14 biomes. For the purposes of our analyses were further grouped these into 7 biome classes: **Tropical rainforests** (including a single mangrove site with analogous climate to adjacent tropical rainforests), **tropical dry forests** (combining broadleaf and coniferous forests), **temperate forests** (combining broadleaf and coniferous forests), **boreal-montane forests** (combining boreal and montane ecosystems), **tropical savannas**, **temperate woodlands** (combining Mediterranean woodlands and temperate grasslands) and **drylands**.

Supplementary Table 3 – Functional trait data sources

Supplementary Table 3: Sources from which functional trait data were obtained for this study. Public records from the TRY database were requested on the 29/10/2020 (trait codes: 4, 14, 3117 and 26 for wood density, leaf nitrogen, SLA and seed mass, respectively). Records from the BIEN database were obtained from version 4.1.1 using the dedicated R package. Publications referenced in the table are cited in full in the main text.

Trait	Units	Source	Link
Leaf nitrogen & SLA	mg g^{-1} & mm^2 mg^{-1}	TRY Plant Trait Database	https://www.try-db.org
Leaf nitrogen & SLA	mg g^{-1} & mm^2 mg^{-1}	AusTraits Database	https://zenodo.org/records/11188867
Leaf nitrogen & SLA	mg g^{-1} & mm^2 mg^{-1}	BIEN Database	https://bien.nceas.ucsb.edu/bien/
Leaf nitrogen & SLA	mg g^{-1} & mm^2 mg^{-1}	China Plant Trait Database	https://esajournals.onlinelibrary.wiley.com/doi/10.1002/ecy.2091
Leaf nitrogen & SLA	mg g^{-1} & mm^2 mg^{-1}	Terrestrial Ecosystem Research Network	https://supersites.tern.org.au/knb/metacat/supersite.949.4/html
Leaf nitrogen & SLA	mg g^{-1} & mm^2 mg^{-1}	Both *et al*. (2019)	https://zenodo.org/records/3247631
Leaf nitrogen & SLA	mg g^{-1} & mm^2 mg^{-1}	Unpublished data from Iran	
Seed mass	g	Kew Gardens Seed Information Database	http://data.kew.org/sid/
Seed mass	g	TRY Plant Trait Database	https://www.try-db.org
Seed mass	g	AusTraits Database	https://doi.org/10.5281/zenodo.3568429
Seed mass	g	Unpublished data from Hainan	
Seed mass	g	Unpublished data from Iran	
Wood density	g cm^{-3}	Global Wood Density Database	https://datadryad.org/stash/dataset/doi:10.5061/dryad.234
Wood density	g cm^{-3}	TRY Plant Trait Database	https://www.try-db.org
Wood density	g cm^{-3}	AusTraits Database	https://zenodo.org/records/11188867
Wood density	g cm^{-3}	Brown *et al*. (1997)	https://www.fao.org/4/w4095e/w4095e0c.htm
Wood density	g cm^{-3}	Díaz *et al*. (2015)	http://www.scielo.org.mx/pdf/mb/v21nspe/v21nspea6.pdf
Wood density	g cm^{-3}	Bradford *et al*. (2014)	https://supersites.tern.org.au/knb/metacat/supersite.174/html
Wood density	g cm^{-3}	Mori *et al*. (2014)	https://academic.oup.com/jpe/article/7/4/356/977001
Wood density	g cm^{-3}	Iida *et al*. (2012)	https://doi.org/10.1111/j.1365-2435.2011.01921.x
Wood density	g cm^{-3}	Unpublished data from Iran	

Supplementary Table 4 – Pairwise comparisons of H_{RESID}, CD_{RESID} and CAR_{RESID} among biomes

Supplementary Table 4: Pairwise differences in size-standardized estimates of tree height (H_{RESID}), crown diameter (CD_{RESID}) and crown aspect ratio (CAR_{RESID}) among biomes. Differences among biomes were tested using one-way ANOVAs with *post hoc* Tukey tests. Biome association explained 33%, 5% and 39% of the variation in H_{RESID}, CD_{RESID} and CAR_{RESID} among species, respectively. Statistically significant differences among biomes are highlighted in bold (P < 0.05).

**Pairwise comparison**	**Difference in H_{RESID}**	***P*-value**	**Difference in CD_{RESID}**	***P*-value**	**Difference in CAR_{RESID}**	***P*-value**
Dryland vs Boreal-montane forest	**-1.40**	**<0.0001**	-0.02	1.000	1.44	**<0.0001**
Temperate forest vs Boreal-montane forest	-0.15	0.522	0.13	0.824	0.30	**0.026**
Temperate forest vs Dryland	**1.24**	**<0.0001**	0.15	0.949	-1.14	**<0.0001**
Temperate woodland vs Boreal-montane forest	**-0.51**	**<0.0001**	0.16	0.720	0.74	**<0.0001**
Temperate woodland vs Dryland	**0.88**	**<0.0001**	0.18	0.897	-0.70	**<0.0001**
Temperate woodland vs Temperate forest	**-0.36**	**<0.0001**	0.03	0.996	0.44	**<0.0001**
Tropical dry forest vs Boreal-montane forest	-0.24	0.081	**0.37**	**0.006**	0.68	**<0.0001**
Tropical dry forest vs Dryland	**1.16**	**<0.0001**	0.38	0.121	-0.76	**<0.0001**
Tropical dry forest vs Temperate forest	-0.09	0.193	**0.24**	**<0.0001**	0.37	**<0.0001**
Tropical dry forest vs Temperate woodland	**0.27**	**<0.0001**	**0.21**	**0.016**	-0.06	0.946
Tropical rainforest vs Boreal-montane forest	-0.04	0.999	0.11	0.911	0.20	0.319
Tropical rainforest vs Dryland	**1.35**	**<0.0001**	0.12	0.976	-1.24	**<0.0001**
Tropical rainforest vs Temperate forest	**0.11**	**<0.0001**	-0.02	0.961	-0.10	**<0.0001**
Tropical rainforest vs Temperate woodland	**0.47**	**<0.0001**	-0.05	0.929	-0.54	**<0.0001**
Tropical rainforest vs Tropical dry forest	**0.20**	**<0.0001**	**-0.26**	**<0.0001**	-0.48	**<0.0001**
Tropical savanna vs Boreal-montane forest	**-0.58**	**<0.0001**	0.34	**0.013**	0.97	**<0.0001**
Tropical savanna vs Dryland	**0.82**	**<0.0001**	0.35	0.187	-0.47	**0.019**
Tropical savanna vs Temperate forest	**-0.43**	**<0.0001**	**0.21**	**<0.0001**	0.67	**<0.0001**
Tropical savanna vs Temperate woodland	-0.07	0.817	**0.17**	**0.043**	0.23	**0.002**
Tropical savanna vs Tropical dry forest	**-0.34**	**<0.0001**	-0.03	0.995	0.29	**<0.0001**
Tropical savanna vs Tropical rainforest	**-0.54**	**<0.0001**	**0.23**	**<0.0001**	0.77	**<0.0001**

Supplementary Table 5 – Variation in H_{RESID}, CD_{RESID} and CAR_{RESID} among plant families

Supplementary Table 5: Mean values of size-standardized estimates of tree height (H_{RESID}), crown diameter (CD_{RESID}) and crown aspect ratio (CAR_{RESID}) of plant families represented by at least 5 species in the analysis (n = 63 families for H_{RESID} and 56 families for CD_{RESID} and CAR_{RESID}). An ANOVA fit without an intercept was used to test whether family-level mean values were significantly different from zero (P < 0.05, highlighted in bold).

Family	H_{RESID}	P-value	CD_{RESID}	P-value	CAR_{RESID}	P-value
Achariaceae	0.049	0.607				
Altingiaceae	-0.024	0.841	-0.091	0.442	-0.092	0.536
Anacardiaceae	**-0.086**	**0.049**	-0.015	0.775	0.117	0.079
Annonaceae	**0.178**	**<0.0001**	**0.119**	**0.013**	-0.062	0.3
Apocynaceae	0.052	0.309	-0.048	0.399	-0.086	0.222
Aquifoliaceae	-0.035	0.642	-0.057	0.475	-0.037	0.711
Araliaceae	0.02	0.805	0.016	0.861	0.065	0.578
Betulaceae	**0.161**	**0.005**	**0.169**	**0.007**	-0.009	0.909
Bignoniaceae	-0.02	0.812	-0.122	0.193	-0.146	0.215
Boraginaceae	-0.033	0.729	-0.067	0.506	0.005	0.969
Burseraceae	**0.19**	**<0.0001**	0.056	0.31	**-0.171**	**0.013**
Calophyllaceae	**0.155**	**0.038**	**0.229**	**0.01**	-0.022	0.84
Cannabaceae	0.019	0.788	0.046	0.536	0.004	0.968
Celastraceae	0.043	0.615	0.034	0.755	-0.077	0.569
Chrysobalanaceae	**0.243**	**0.01**	**0.216**	**0.047**	-0.027	0.84
Clusiaceae	0.066	0.393	0.158	0.075	0.091	0.411
Combretaceae	**-0.298**	**<0.0001**	**0.165**	**0.008**	**0.495**	**<0.0001**
Cornaceae	0.106	0.331	0.062	0.604	0.004	0.978
Cunoniaceae	0.113	0.208				
Cupressaceae	**-0.277**	**<0.0001**	**-0.177**	**0.027**	0.097	0.333
Dipterocarpaceae	**0.265**	**<0.0001**	0.059	0.192	**-0.236**	**<0.0001**
Ebenaceae	0.001	0.987	-0.09	0.174	-0.094	0.259
Elaeocarpaceae	0.089	0.104	**-0.181**	**0.031**	-0.092	0.382
Ericaceae	**-0.277**	**0.004**	0.072	0.505	**0.273**	**0.044**
Euphorbiaceae	**0.085**	**0.013**	0.025	0.524	-0.075	0.128
Fabaceae	**-0.095**	**<0.0001**	**0.144**	**<0.0001**	**0.231**	**<0.0001**
Fagaceae	**-0.123**	**<0.0001**	-0.015	0.593	**0.098**	**0.005**
Juglandaceae	0.004	0.966	0.128	0.202	0.113	0.369
Lamiaceae	-0.156	0.066	**-0.291**	**0.015**	-0.254	0.087
Lauraceae	**0.087**	**0.001**	**-0.157**	**<0.0001**	**-0.158**	**<0.0001**
Lecythidaceae	**0.149**	**0.023**	0.031	0.69	**-0.261**	**0.006**
Loganiaceae	**-0.452**	**<0.0001**	**0.222**	**0.041**	**0.671**	**<0.0001**
Magnoliaceae	-0.041	0.628	**-0.226**	**0.024**	-0.153	0.222
Malvaceae	-0.003	0.926	-0.047	0.178	-0.047	0.283
Melastomataceae	0.094	0.356	0.143	0.187	-0.001	0.997
Meliaceae	**0.086**	**0.034**	0.034	0.459	-0.04	0.48
Moraceae	-0.036	0.408	-0.074	0.155	-0.013	0.844
Myristicaceae	**0.257**	**<0.0001**	**0.15**	**0.02**	-0.134	0.095

Myrtaceae	**-0.052**	**0.007**	**-0.099**	**0.006**	-0.085	0.064
Nothofagaceae	-0.069	0.565				
Nyctaginaceae	-0.147	0.221				
Olacaceae	**0.173**	**0.033**	**0.18**	**0.033**	0.016	0.882
Oleaceae	-0.016	0.847	-0.075	0.373	-0.082	0.432
Pentaphylacaceae	-0.032	0.705	-0.142	0.11	-0.101	0.361
Phyllanthaceae	-0.028	0.564	0.017	0.753	0.05	0.474
Pinaceae	**-0.116**	**<0.0001**	**-0.182**	**<0.0001**	**-0.138**	**0.002**
Podocarpaceae	-0.07	0.432	-0.177	0.059	-0.112	0.341
Primulaceae	-0.07	0.432	**-0.254**	**0.007**	-0.203	0.084
Proteaceae	0.087	0.148				
Putranjivaceae	**0.193**	**0.017**	**0.216**	**0.01**	-0.034	0.744
Rhamnaceae	0.047	0.602	0.066	0.544	0.17	0.211
Rosaceae	-0.111	0.089	-0.033	0.644	0.125	0.16
Rubiaceae	-0.002	0.967	**-0.109**	**0.034**	**-0.135**	**0.034**
Rutaceae	0.074	0.11	-0.082	0.266	-0.049	0.591
Salicaceae	**0.115**	**0.036**	0.021	0.74	-0.083	0.286
Sapindaceae	0.049	0.195	0.012	0.795	-0.008	0.892
Sapotaceae	**0.123**	**0.005**	0.062	0.246	-0.108	0.105
Symplocaceae	-0.092	0.235	-0.095	0.214	0.009	0.925
Theaceae	-0.059	0.488	**-0.198**	**0.019**	-0.137	0.193
Thymelaeaceae	0.105	0.382				
Ulmaceae	-0.136	0.109	**0.24**	**0.007**	**0.391**	**<0.0001**
Urticaceae	**0.211**	**0.009**	0.112	0.232	-0.131	0.265
Vochysiaceae	0.055	0.647				

Supplementary Table 6 – Variation in H_{RESID}, CD_{RESID} and CAR_{RESID} among plant genera

Supplementary Table 6: Mean values of size-standardized estimates of tree height (H_{RESID}), crown diameter (CD_{RESID}) and crown aspect ratio (CAR_{RESID}) of plant genera represented by at least 5 species in the analysis (n = 86 genera for H_{resid} and 60 genera for CD_{resid} and CAR_{resid}). An ANOVA fit without an intercept was used to test whether genus-level mean values were significantly different from zero (P < 0.05, highlighted in bold).

Genus	H_{resid}	*P*-value	CD_{resid}	*P*-value	CAR_{resid}	*P*-value
Abies	**-0.168**	**0.017**	**-0.242**	**0.003**	**-0.238**	**0.015**
Acacia	**-0.314**	**<0.001**	**0.352**	**0.001**	**0.709**	**<0.001**
Acer	0.044	0.466	0.059	0.368	-0.001	0.989
Aglaia	0.105	0.333	0.007	0.949	-0.097	0.461
Alangium	0.133	0.221				
Albizia	**-0.231**	**0.004**	0.084	0.305	**0.318**	**0.001**
Alnus	0.169	0.121				
Alstonia	0.103	0.342				
Archidendron	-0.019	0.863				
Artocarpus	0.045	0.596	-0.115	0.249	-0.152	0.205
Aspidosperma	**0.259**	**0.017**	-0.007	0.947	-0.249	0.058
Beilschmiedia	**0.143**	**0.028**	-0.127	0.144	-0.187	0.071
Betula	0.142	0.065	0.138	0.137	-0.038	0.731
Brachystegia	**-0.307**	**0.005**	**0.329**	**0.003**	**0.653**	**<0.001**
Caesalpinia	-0.183	0.092				
Calophyllum	0.146	0.072	**0.217**	**0.019**	-0.017	0.875
Canarium	0.151	0.1	-0.119	0.277	**-0.258**	**0.05**
Carya	0.135	0.214				
Castanopsis	-0.108	0.086	**-0.151**	**0.027**	-0.008	0.918
Cecropia	0.209	0.054				
Celtis	0.074	0.312	0.101	0.219	-0.017	0.859
Chrysophyllum	0.126	0.202	**0.308**	**0.005**	0.173	0.187
Cinnamomum	-0.013	0.873	**-0.255**	**0.011**	-0.202	0.092
Combretum	**-0.506**	**<0.001**	**0.211**	**0.035**	**0.797**	**<0.001**
Cordia	0.006	0.95	-0.066	0.55	-0.036	0.784
Corymbia	-0.09	0.107				
Croton	0.155	0.091	0.173	0.084	0.011	0.926
Cryptocarya	**0.121**	**0.026**	**-0.186**	**0.023**	-0.13	0.182
Diospyros	0.001	0.985	-0.09	0.14	-0.094	0.201
Dipterocarpus	**0.283**	**0.004**				
Drypetes	**0.193**	**0.008**	**0.216**	**0.005**	-0.034	0.712
Dysoxylum	0.09	0.365				
Elaeocarpus	0.073	0.217	**-0.288**	**0.002**	-0.158	0.153
Endiandra	**0.303**	**<0.001**				
Eucalyptus	**-0.115**	**<0.001**	**-0.212**	**0.004**	-0.013	0.883
Fagus	0.092	0.398	**0.306**	**0.005**	0.164	0.211
Ficus	**-0.162**	**0.021**	**-0.257**	**0.002**	-0.025	0.794
Flindersia	**0.349**	**<0.001**				
Fraxinus	0.013	0.9	0.081	0.419	0.023	0.847

Garcinia	0.086	0.287	0.191	0.057	0.105	0.379
Glochidion	0.097	0.326				
Heritiera	0.129	0.237				
Hopea	0.188	0.058	-0.105	0.34	**-0.268**	**0.041**
Hydnocarpus	0.005	0.965				
Ilex	-0.035	0.607	-0.057	0.438	-0.037	0.674
Inga	**0.246**	**0.024**	**0.262**	**0.017**	0.009	0.944
Knema	**0.3**	**0.001**	**0.258**	**0.019**	-0.064	0.623
Larix	0.136	0.169	-0.126	0.249	-0.249	0.057
Licania	**0.283**	**0.009**				
Lindera	-0.045	0.65	-0.179	0.073	-0.125	0.295
Lithocarpus	-0.01	0.872	-0.097	0.102	-0.086	0.226
Litsea	0.118	0.079	-0.052	0.499	-0.062	0.504
Lonchocarpus	-0.043	0.637	0.07	0.521	0.16	0.221
Macaranga	0.111	0.113	-0.04	0.668	-0.161	0.147
Machilus	-0.101	0.212	**-0.275**	**0.001**	-0.173	0.078
Magnolia	-0.076	0.348	**-0.295**	**0.003**	-0.157	0.188
Mallotus	-0.002	0.985				
Manilkara	0.055	0.614				
Melaleuca	**-0.43**	**<0.001**				
Myristica	**0.231**	**0.02**				
Neolitsea	-0.033	0.702	**-0.296**	**0.003**	**-0.255**	**0.034**
Nothofagus	-0.069	0.524				
Ocotea	**0.199**	**0.044**				
Ormosia	0.038	0.677	**-0.2**	**0.046**	-0.22	0.066
Palaquium	**0.268**	**0.014**				
Picea	0.058	0.45	**-0.299**	**0.001**	**-0.412**	**<0.001**
Pinus	**-0.202**	**<0.001**	**-0.14**	**0.002**	0.005	0.924
Populus	0.133	0.148	0.058	0.529	-0.078	0.481
Pouteria	**0.249**	**0.007**	0.053	0.57	-0.213	0.054
Protium	**0.278**	**0.003**	0.172	0.117	-0.106	0.417
Prunus	0.037	0.711	0.07	0.521	0.083	0.529
Pterocarpus	**-0.197**	**0.032**	**0.302**	**0.003**	**0.508**	**<0.001**
Quercus	**-0.176**	**<0.001**	0.012	0.719	**0.176**	**<0.001**
Schima	-0.016	0.886	-0.106	0.335	-0.093	0.475
Shorea	**0.286**	**<0.001**	0.008	0.897	**-0.35**	**<0.001**
Sloanea	0.128	0.198				
Sterculia	0.185	0.089	-0.024	0.829	-0.206	0.116
Strychnos	**-0.452**	**<0.001**	**0.222**	**0.027**	**0.671**	**<0.001**
Symplocos	-0.092	0.189	-0.095	0.179	0.009	0.914
Syzygium	0.063	0.071	-0.062	0.17	-0.097	0.076
Terminalia	**-0.188**	**0.01**	0.151	0.105	**0.286**	**0.01**
Trichilia	0.159	0.143	**0.363**	**0.001**	0.22	0.093
Ulmus	-0.166	0.094	**0.222**	**0.027**	**0.416**	**0.001**
Vachellia	**-0.764**	**<0.001**	0.068	0.46	**0.969**	**<0.001**
Virola	**0.276**	**0.005**				
Vitex	-0.207	0.057				

Supplementary Fig. 1 – Size-standardised estimates of tree height and crown size

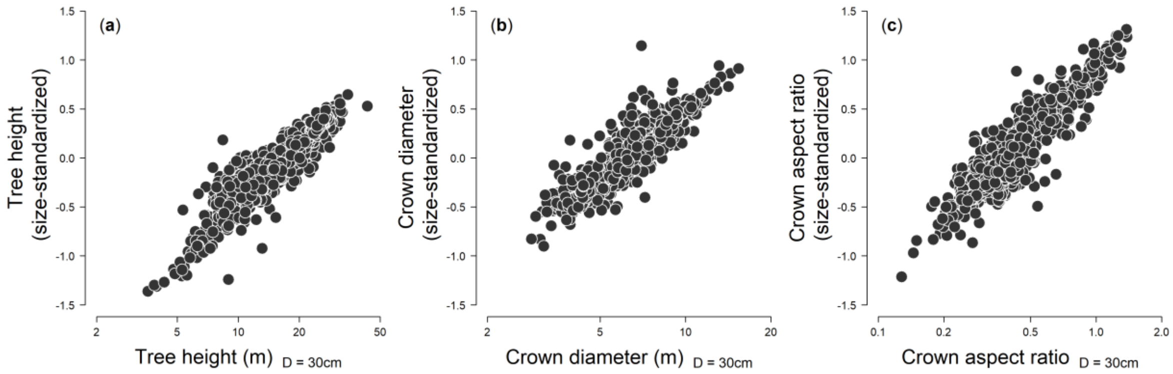

Supplementary Fig. 1: Comparison between species' size-standardized estimates of (**a**) tree height, (**b**) crown diameter and (**c**) crown aspect ratio generated using the model residual approach described in the main text and predicted values of all three attributes estimated for a tree of fixed size (stem diameter, D = 30 cm). Pearson's correlation coefficients for the comparisons shown above ranged between 0.91–0.93 ($P < 0.0001$ in all cases).

Supplementary Fig. 2 – Comparing power-law and Michaelis-Menten height–diameter models

To determine how much size-standardised estimates of tree height (H_{RESID} and $H_{D=30}$) are influenced by the choice of functional form used to model height–diameter relationships, we compared two alternative functional forms [2]: a power-law model (as described in the main text) and a saturating Michaelis-Menten function, where tree height (H) is expressed as the following function of stem diameter (D): $\log(H) = log\left(\frac{h_{max} \times D}{k+D}\right)$, where h_{max} and k are parameters to be estimated from the data.

To robustly compare these two alternative functional forms, we used a subset 754 of well sampled species in our datasets (minimum sample size = 50 trees per species; minimum stem diameter range = 30 cm). For each species we took a random subset of 50 individuals to ensure species with larger sample sizes would not dominate the signal. We then used the same approach described in the Methods of the main text to estimate H_{RESID} and $H_{D=30}$ for each species using both a power-law and a Michaelis-Menten function (see main text for how to access data and R code to replicate this analysis). We found that both estimates of H_{RESID} ($\rho = 0.97$) $H_{D=30}$ ($\rho = 0.93$) derived from a power-law and a Michaelis-Menten function were strongly correlated (Supplementary Fig. 2). However, of the two functional forms the power-law model fit the data better both when modelling the entire dataset together to estimate H_{RESID} (RMSE = 5.4 m and 5.8 m for power-law and Michaelis-Menten, respectively) and when accounting for variation in allometric scaling relationships among species to estimate $H_{D=30}$ (RMSE = 3.5 m and 3.9 m for power-law and Michaelis-Menten, respectively). Based on this we opted to model height–diameter relationships using a power-law function.

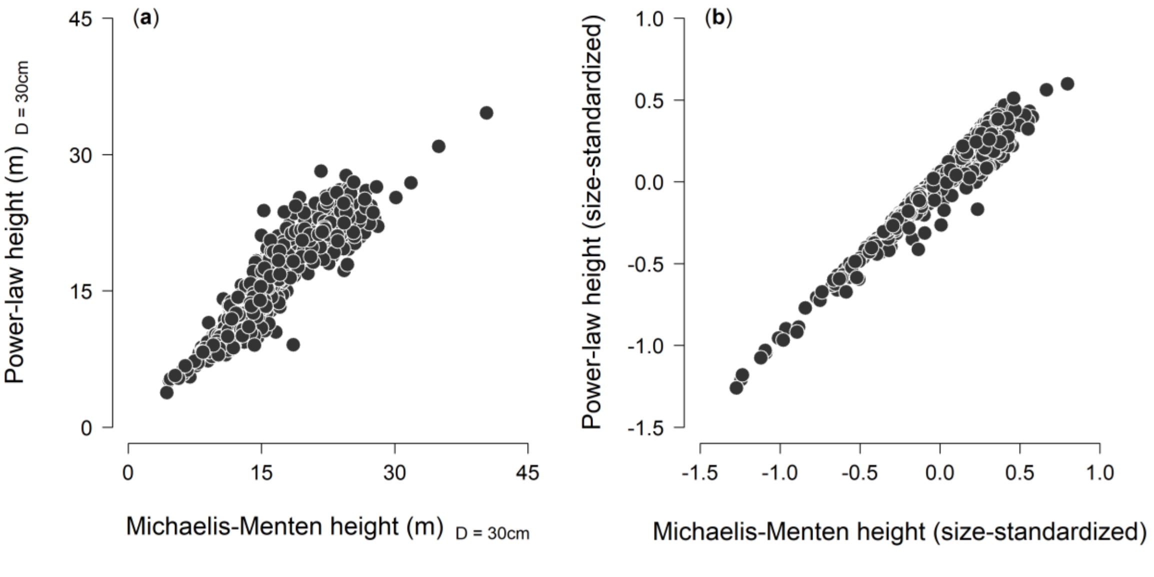

Supplementary Fig. 2: Comparison between species' estimates of (**a**) $H_{D=30}$ and (**b**) H_{RESID} when using a power-law and a Michaelis-Menten function to model height–diameter relationships across 754 well sampled species in our database.

Supplementary Fig. 3 – Correlations among model predictors

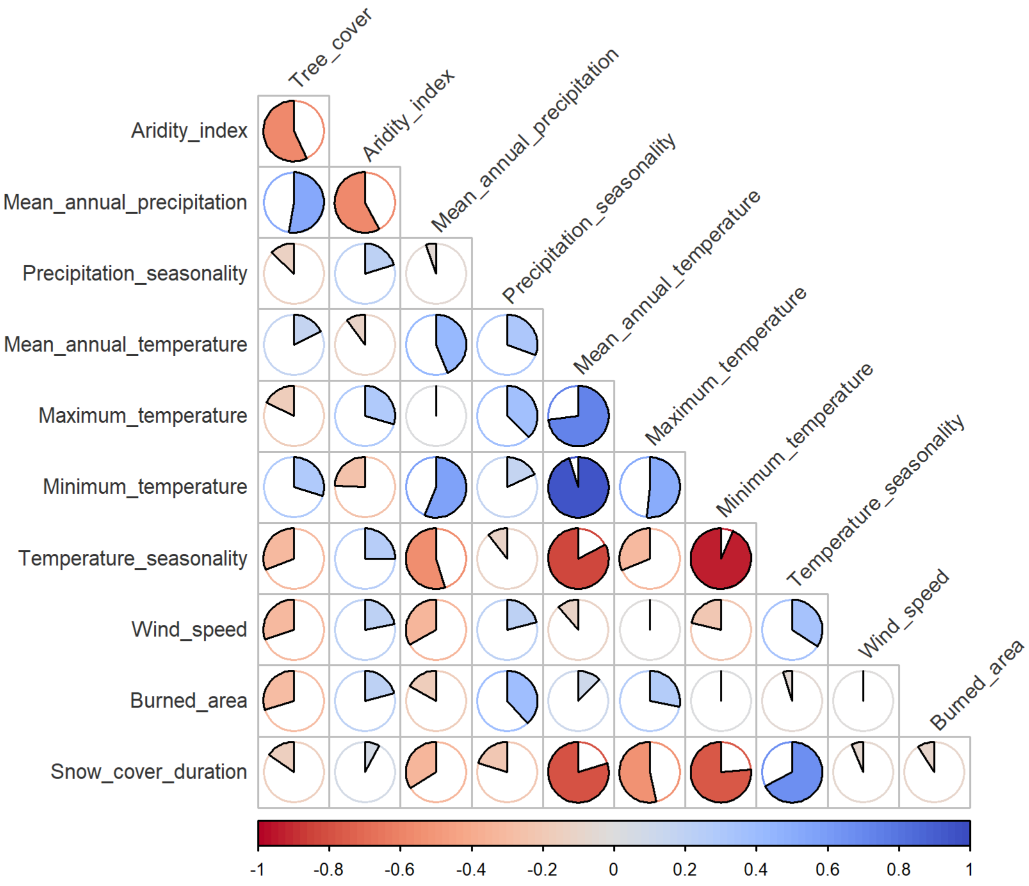

Supplementary Fig. 3: Pearson's correlation coefficients among bioclimatic and disturbance predictors used in the models described in the main text. Data sources for each bioclimatic and disturbance attribute are reported in Supplementary Table 2.

Supplementary Fig. 4 – MODIS-derived tree cover as a proxy for local competitive environment

We used data from 851 geo-located forest plots spanning major forested and non-forested biomes to evaluate how well MODIS-derived estimates of tree cover compare to field-estimated proxies of competitive environment – specifically forest basal area. Tree cover was estimated from MODIS at 500 m resolution for the year 2008 [3]. Plot-level basal area values were obtained from global and regional databases, including the Forest Observation System (www.forest-observation-system.net) [4], the FunDivEUROPE network [5], Australia's Biomass Plot Library (www.auscover.org.au/datasets/biomass-plot-library), and a network of forest plots distributed across the Gola Rainforest National Park in Sierra Leone [6]. Using these data, we fit a binomial GLM relating variation in tree cover (scaled between 0–1) to plot-level estimates of basal area (log-transformed). This revealed a significantly positive relationship between tree cover and forest basal area (P < 0.0001, McFadden's pseudo R^2 = 0.48; Supplementary Fig. 4), suggesting that MODIS-derived estimates of tree cover provide a robust proxy of a tree's local competitive environment.

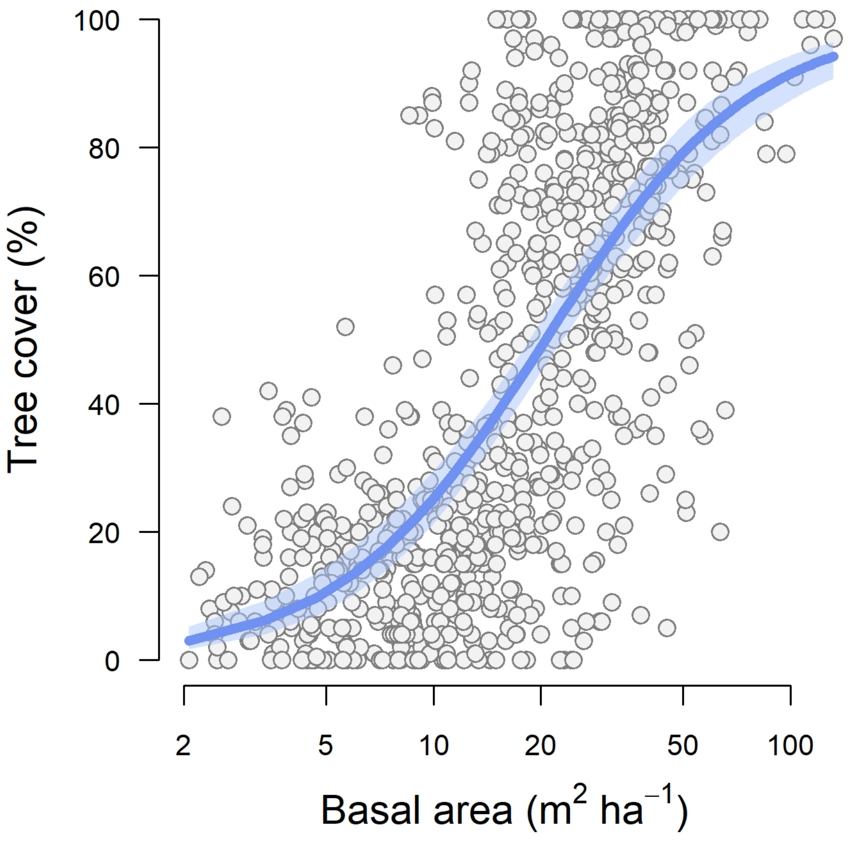

Supplementary Fig. 4: Relationship between MODIS-derived estimates of tree cover and field-measured values of basal area across 851 forest plots. A line of best fit with shaded 95% confidence intervals from a binomial GLM is shown in blue.

Supplementary Fig. 5 – Variation in crown architectural types among gymnosperms and angiosperms

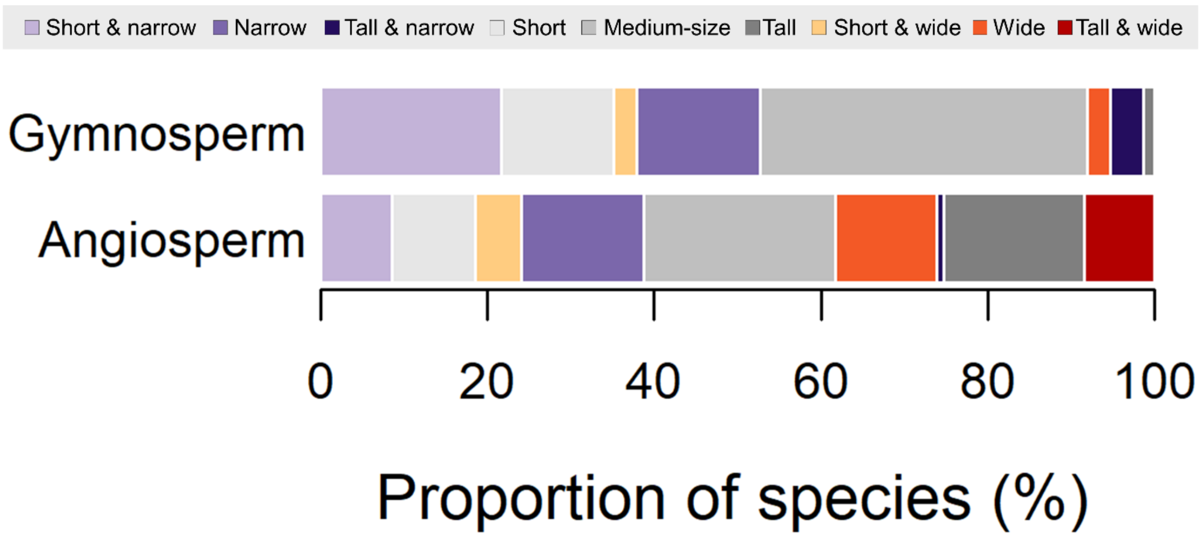

Supplementary Fig. 5: Distribution of tree crown architectural types among gymnosperms and angiosperms for the 1309 tree species for which both height and crown size were measured. Tree species were grouped into one of nine architectural types based on their size-standardized height and crown diameter values (see Fig. 2 in the main text for details).

Supplementary Fig. 6 – Interactive effects of aridity and temperature on tree crown architecture

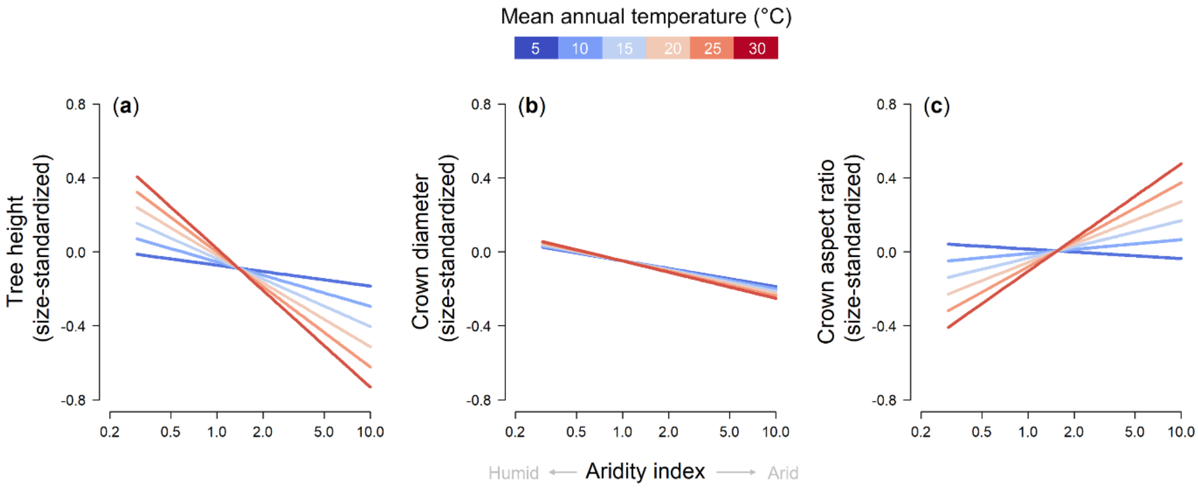

Supplementary Fig. 6: Interactive effects of aridity and mean annual temperature on tree crown architecture. Lines show how species' size-standardized height (**a**), crown diameter (**b**) and crown aspect ratio (**c**) values are predicted to vary along an aridity gradient for different levels of mean annual temperature. Fitted lines were generated from the phylogenetic generalised least squares models while keeping all other predictors fixed at their mean value.

References

1. Smith, S. A. & Brown, J. W. Constructing a broadly inclusive seed plant phylogeny. *Am J Bot* **105**, 302–314 (2018).

2. Cano, I. M., Muller-Landau, H. C., Joseph Wright, S., Bohlman, S. A. & Pacala, S. W. Tropical tree height and crown allometries for the Barro Colorado Nature Monument, Panama: A comparison of alternative hierarchical models incorporating interspecific variation in relation to life history traits. *Biogeosciences* **16**, 847–862 (2019).

3. Kobayashi, T., Tsend-Ayush, J. & Tateishi, R. A new global tree-cover percentage map using MODIS data. *Int J Remote Sens* **37**, 969–992 (2016).

4. Schepaschenko, D. *et al.* The Forest Observation System, building a global reference dataset for remote sensing of forest biomass. *Sci Data* **6**, 198 (2019).

5. Baeten, L. *et al.* A novel comparative research platform designed to determine the functional significance of tree species diversity in European forests. *Perspect Plant Ecol Evol Syst* **15**, 281–291 (2013).

6. Jucker, T. *et al.* Drivers of aboveground wood production in a lowland tropical forest of West Africa: teasing apart the roles of tree density, tree diversity, soil phosphorus, and historical logging. *Ecol Evol* **6**, 4004–4017 (2016).
