# Supplementary material for: The global spectrum of tree crown architecture
Source: Nat Commun. 2025 May 26;16:4876. doi: 10.1038/s41467-025-60262-x (PMC12106748; doi:10.1038/s41467-025-60262-x)
Supplement: Supplementary file 2 — Reporting Summary [file 41467_2025_60262_MOESM2_ESM.pdf]

nature portfolio

Corresponding author(s): Tommaso Jucker

Last updated by author(s): Apr 13, 2025

Reporting Summary

Nature Portfolio wishes to improve the reproducibility of the work that we publish. This form provides structure for consistency and transparency in reporting. For further information on Nature Portfolio policies, see our Editorial Policies and the Editorial Policy Checklist.

Statistics

For all statistical analyses, confirm that the following items are present in the figure legend, table legend, main text, or Methods section.

n/a	Confirmed	
☐	☒	The exact sample size (*n*) for each experimental group/condition, given as a discrete number and unit of measurement
☒	☐	A statement on whether measurements were taken from distinct samples or whether the same sample was measured repeatedly
☐	☒	The statistical test(s) used AND whether they are one- or two-sided<br>*Only common tests should be described solely by name; describe more complex techniques in the Methods section.*
☐	☒	A description of all covariates tested
☐	☒	A description of any assumptions or corrections, such as tests of normality and adjustment for multiple comparisons
☐	☒	A full description of the statistical parameters including central tendency (e.g. means) or other basic estimates (e.g. regression coefficient) AND variation (e.g. standard deviation) or associated estimates of uncertainty (e.g. confidence intervals)
☐	☒	For null hypothesis testing, the test statistic (e.g. *F*, *t*, *r*) with confidence intervals, effect sizes, degrees of freedom and *P* value noted<br>*Give P values as exact values whenever suitable.*
☒	☐	For Bayesian analysis, information on the choice of priors and Markov chain Monte Carlo settings
☐	☒	For hierarchical and complex designs, identification of the appropriate level for tests and full reporting of outcomes
☐	☒	Estimates of effect sizes (e.g. Cohen's *d*, Pearson's *r*), indicating how they were calculated

Our web collection on statistics for biologists contains articles on many of the points above.

Software and code

Policy information about availability of computer code

Data collection	All data processing and analysis were carried out using the statistical software R (v 4.2.2)
Data analysis	All data processing and analysis were carried out using the statistical software R (v 4.2.2)

For manuscripts utilizing custom algorithms or software that are central to the research but not yet described in published literature, software must be made available to editors and reviewers. We strongly encourage code deposition in a community repository (e.g. GitHub). See the Nature Portfolio guidelines for submitting code & software for further information.

Data

Policy information about availability of data

All manuscripts must include a data availability statement. This statement should provide the following information, where applicable:

- Accession codes, unique identifiers, or web links for publicly available datasets
- A description of any restrictions on data availability
- For clinical datasets or third party data, please ensure that the statement adheres to our policy

Data and R code supporting the results of this study are publicly archived on Zenodo (https://doi.org/10.5281/zenodo.14217401). Allometry data from the TALLO database can be accessed here: https://zenodo.org/records/6637599. ICP Forests allometry data are available here: http://icp-forests.net/page/data-requests. Alberta PSP allometry data are available here: https://www.alberta.ca/permanent-sample-plots-program.aspx.

Research involving human participants, their data, or biological material

Policy information about studies with human participants or human data. See also policy information about sex, gender (identity/presentation), and sexual orientation and race, ethnicity and racism.

Reporting on sex and gender	NA
Reporting on race, ethnicity, or other socially relevant groupings	NA
Population characteristics	NA
Recruitment	NA
Ethics oversight	NA

Note that full information on the approval of the study protocol must also be provided in the manuscript.

Field-specific reporting

Please select the one below that is the best fit for your research. If you are not sure, read the appropriate sections before making your selection.

☐ Life sciences ☐ Behavioural & social sciences ☒ Ecological, evolutionary & environmental sciences

For a reference copy of the document with all sections, see nature.com/documents/nr-reporting-summary-flat.pdf

Ecological, evolutionary & environmental sciences study design

All studies must disclose on these points even when the disclosure is negative.

Study description	We compiled georeferenced records of individual trees for which stem diameter (D, cm), height (H, m) and/or crown diameter (CD, m) were measured. These are described in detail here: https://onlinelibrary.wiley.com/doi/full/10.1111/gcb.16302. We combined these records with environmental and trait data derived from public databases and the literature . For a detailed description of these data see Tables S2-3 in supporting information.
Research sample	A total of 374,888 individual tree records were used to estimated size-standardized estimates of tree height, crown diameter and crown aspect ratio for 1914 tree species.
Sampling strategy	We only retained species for which at least 10 trees were sampled within the same biome and for which the stem diameter range between the smallest and largest measured tree was at least 20 cm.
Data collection	Measurements of tree D, H and CD were recorded in the filed as part of forest plot inventories through variety of projects led by the co-authors of this study.
Timing and spatial scale	Data were acquired globally over the course of forest plot inventories carried out between the 1980s to present.
Data exclusions	NA
Reproducibility	All data and R code needed to replicate the results of our study are provided as supporting information to this submission.
Randomization	NA
Blinding	The data used in this study were compiled from previously published studies as described here: https://onlinelibrary.wiley.com/doi/full/10.1111/gcb.16302.

Did the study involve field work? ☐ Yes ☒ No

Reporting for specific materials, systems and methods

We require information from authors about some types of materials, experimental systems and methods used in many studies. Here, indicate whether each material, system or method listed is relevant to your study. If you are not sure if a list item applies to your research, read the appropriate section before selecting a response.

Materials & experimental systems

n/a	Involved in the study
☒	☐ Antibodies
☒	☐ Eukaryotic cell lines
☒	☐ Palaeontology and archaeology
☒	☐ Animals and other organisms
☒	☐ Clinical data
☒	☐ Dual use research of concern
☒	☐ Plants

Methods

n/a	Involved in the study
☒	☐ ChIP-seq
☒	☐ Flow cytometry
☒	☐ MRI-based neuroimaging

Plants

Seed stocks	NA
Novel plant genotypes	NA
Authentication	NA
